# Supplementary material for: Novel Putative Positive Modulators of α4β2 nAChRs Potentiate Nicotine Reward-Related Behavior
Source: Molecules. 2021 Aug 7;26(16):4793. doi: 10.3390/molecules26164793 (PMC8398432; doi:10.3390/molecules26164793)
Supplement: Supplementary file 1 [file molecules-26-04793-s001.zip › molecules-1329492-supplementary.pdf]

Supplemental Discussion

Article

Novel Putative Positive Modulators of $\alpha 4\beta 2$ nAChRs Potentiate Nicotine Reward-Related Behavior

[1] Department of Biomedical Sciences, Joan C Edwards School of Medicine, Marshall University,
Huntington, WV 25703, USA; cooper394@live.marshall.edu (S.Y.C.);
akers103@live.marshall.edu (A.T.A.);
journigan@marshall.edu (V.B.J.)
[2] Department of Pharmaceutical Sciences, School of Pharmacy, Marshall University,
Huntington, WV 25701, USA
* Correspondence: hendersonbr@marshall.edu

Preliminary pharmacokinetic assays show that VBJ104 exhibits a half-life ($T_{1/2}$) of 11.3 minutes in mouse liver microsomes and an intrinsic clearance (CL_{int}) of 0.122 mL/min/mg (see *Supplemental Material*, Table S2) longer than the reported $T_{1/2}$ of nicotine in mice (6-7 min) [1]. Additionally, VBJ104 exhibited a low MDR1-MDCK permeability efflux ratio (Table S1) [2]. We must also consider nicotine's ability to enter cells and their organelles [3-5]. It is possible that VBJ104 may also interact with nicotine to alter its pharmacological chaperoning effect. While these questions may be interesting from the pharmacological standpoint, it is unlikely these compounds will aid in the movement toward novel small molecules useful for nicotine cessation. We did not test the effects of these ligands as potential blockers of the sensory impact of mentholated cigarette smoke upon respiratory exposure, and so it remains to be determined whether TRPM8 antagonists may prevent enhanced self-administration of oral nicotine.

Supplemental Methods

MDR1-MDCK Permeability. Pharmacokinetic experiments with compounds were performed by Absorption Systems (Exton, PA, USA). MDR1-MDCK cell monolayers were grown to confluence on collagen-coated, microporous membranes in 12-well assay plates. The permeability assay buffer was Hanks' balanced salt solution containing 10 mM HEPES and 15 mM glucose at a pH of 7.4. The buffer in the receiver chamber also contained 1% bovine serum albumin. The dosing solution concentration was 5

μM of test article in the assay buffer. Cell monolayers were dosed on the apical side (A-to-B) or basolateral side (B-to-A) and incubated at 37°C with 5% CO_2 in a humidified incubator. Samples were taken from the donor

chambers at 0 and 120 minutes; samples were taken from the receiver chambers at 120 minutes. Each determination was performed in duplicate. The flux of lucifer yellow was also measured post-experimentally for each monolayer to ensure no damage was inflicted to the cell monolayers during the flux period. All samples were assayed by LC-MS/MS using electrospray ionization. Analytical conditions were carried out as previously described (REF:Journigan ACS CN 2020). The apparent permeability (P_{app}) and percent recovery were calculated as follows:

$$P_{app} = (dC_r / dt) \times V_r / (A \times C_0) \quad (1)$$

$$\text{Percent Recovery} = 100 \times ((V_r \times C_r^{final}) + (V_d \times C_d^{final}))/(V_d \times C_0) \quad (2)$$

Where,

dC_r / dt is the slope of the cumulative receiver concentration versus time in M s^{-1};
V_r is the volume of the receiver compartment in cm^3;
V_d is the volume of the donor compartment in cm^3;
A is the area of the insert (1.13 cm^2 for 12-well);
C_0 is the measured concentration of the donor chamber at time 0 in μM;
C_r^{final} is the cumulative receiver concentration in μM at the end of the incubation period;
C_d^{final} is the concentration of the donor in μM at the end of the incubation period.

Efflux ratio (ER) is defined as P_{app} (B-to-A) / P_{app} (A-to-B).

Brain Penetration Potential Classification:

P_{app} (A-to-B) ≥ 3.0 and ER < 3.0: **High**
P_{app} (A-to-B) ≥ 3.0 and 10 > ER ≥ 3.0: **Moderate**
P_{app} (A-to-B) ≥ 3.0 and ER ≥ 10, or P_{app} (A-to-B) < 3.0: **Low**

Mouse Liver Microsome Assays. These assays were carried out as detailed previously [6].

Supplemental Tables

**Supplemental Table S1. MDR1-MDCK Permeability**							
	**Direction**	**Recovery (%)**	**P_{app} (10^{-6} cm/s)**			**Efflux Ratio**	**Brain Penetration Classification**
			R1	R2	average		
**VBJ104**	A-to-B	70.8	1.90	1.67	1.79	0.685	Low
	B-to-A	86.9	0.919	1.53	1.22		
**digoxin**	A-to-B		0.0612			299	
	B-to-A		18.3				

**Supplemental Table S2. Stability in Mouse Liver Microsomes**								
	**Species**	**% Remaining of Initial**					**Half-life (min)**	**CL_{int}[a] (ml/min/mg protein)**
		0 min	10 min	20 min	30 min	60 min		
**VBJ104**	mouse	100	40.8	30.7	22.5	10.3	11.3	0.122
**testosterone**	mouse						4.58	0.302

[a] Calculated according to (REF: Journigan ACS CN 2020).

References

1. Matta, S.G., D.J. Balfour, N.L. Benowitz, R.T. Boyd, J.J. Buccafusco, A.R. Caggiula, C.R. Craig, A.C. Collins, M.I. Damaj, E.C. Donny, P.S. Gardiner, S.R. Grady, U. Heberlein, S.S. Leonard, E.D. Levin, R.J. Lukas, A. Markou, M.J. Marks, S.E. McCallum, N. Parameswaran, K.A. Perkins, M.R.

Picciotto, M. Quik, J.E. Rose, A. Rothenfluh, W.R. Schafer, I.P. Stolerman, R.F. Tyndale, J.M. Wehner, and J.M. Zirger, *Guidelines on nicotine dose selection for in vivo research.* Psychopharm, 2007. **190**(3): p. 269-319.

2. Dolghih, E. and M.P. Jacobson, *Predicting efflux ratios and blood-brain barrier penetration from chemical structure: combining passive permeability with active efflux by P-glycoprotein.* ACS Chem Neurosci, 2013. **4**(2): p. 361-7.
3. Shivange, A.V., P.M. Borden, A.K. Muthusamy, A.L. Nichols, K. Bera, H. Bao, I. Bishara, J. Jeon, M.J. Mulcahy, B. Cohen, S.L. O'Riordan, C. Kim, D.A. Dougherty, E.R. Chapman, J.S. Marvin, L.L. Looger, and H.A. Lester, *Determining the pharmacokinetics of nicotinic drugs in the endoplasmic reticulum using biosensors.* J Gen Physiol, 2019. **151**(6): p. 738-757.
4. Kuryatov, A., J. Luo, J. Cooper, and J. Lindstrom, *Nicotine acts as a pharmacological chaperone to up-regulate human a4b2 acetylcholine receptors.* Mol Pharmacol, 2005. **68**(6): p. 1839-51.
5. Srinivasan, R., R. Pantoja, F.J. Moss, E.D.W. Mackey, C. Son, J. Miwa, and H.A. Lester *Nicotine upregulates α4β2 nicotinic receptors and ER exit sites via stoichiometry-dependent chaperoning.* J. Gen .Physiol., 2011. **137**: p. 59-79.
6. Journigan, V.B., Z. Feng, S. Rahman, Y. Wang, A. Amin, C.E. Heffner, N. Bachtel, S. Wang, S. Gonzalez-Rodriguez, A. Fernandez-Carvajal, G. Fernandez-Ballester, J.K. Hilton, W.D. Van Horn, A. Ferrer-Montiel, X.Q. Xie, and T. Rahman, *Structure-Based Design of Novel Biphenyl Amide Antagonists of Human Transient Receptor Potential Cation Channel Subfamily M Member 8 Channels with Potential Implications in the Treatment of Sensory Neuropathies.* ACS Chem Neurosci, 2020. **11**(3): p. 268-290.
